# Minimum Vertex-type Sequence Indexing for Clusters on Square Lattice

**DOI:** 10.1038/s41598-017-00398-z

**Published:** 2017-03-24

**Authors:** Longguang Liao, Yu-Jun Zhao, Zexian Cao, Xiao-Bao Yang

**Affiliations:** 1 Department of Physics, South China University of Technology, Guangzhou, 510640 China; 2 Key Laboratory of Advanced Energy Storage Materials of Guangdong Province, South China University of Technology, Guangzhou, 510640 China; 3 Institute of Physics, Chinese Academy of Sciences, Beijing, 100190 China

## Abstract

An effective indexing scheme for clusters that enables fast structure comparison and congruence check is desperately desirable in the field of mathematics, artificial intelligence, materials science, etc. Here we introduce the concept of minimum vertex-type sequence for the indexing of clusters on square lattice, which contains a series of integers each labeling the vertex type of an atom. The minimum vertex-type sequence is orientation independent, and it builds a one-to-one correspondence with the cluster. By using minimum vertex-type sequence for structural comparison and congruence check, only one type of data is involved, and the largest amount of data to be compared is *n* pairs, *n* is the cluster size. In comparison with traditional coordinate-based methods and distance-matrix methods, the minimum vertex-type sequence indexing scheme has many other remarkable advantages. Furthermore, this indexing scheme can be easily generalized to clusters on other high-symmetry lattices. Our work can facilitate cluster indexing and searching in various situations, it may inspire the search of other practical indexing schemes for handling clusters of large sizes.

## Introduction

Clusters containing a few to several thousands of atoms serve to bridge the gap between an isolated atom and the bulk counterpart^1–3^. They may exhibit peculiar physical and chemical properties that are probably not to be observed in the bulk materials. Small clusters are anticipated to exhibit strongly size-dependent features, while those intermediate- and large-sized clusters might exhibit a smoothly varying behavior which approaches the bulk limit^3–5^. As the number of atoms increases, the crystal fragments were found to be more stable^5, 6^. Thus, the congruence-check of the crystal fragments is important, while there is a lack of a high-efficient method for distinguishing the clusters containing bonds of same lengths.

The size and structure are the fundamental factors in determining the properties of a cluster, they are thus of great concern in the study and application of clusters. The conventional experimental methods for structural determination are hardly able to directly obtain the atomic configuration of clusters^7–11^, as collaborating with theoretical computation is usually a necessity^1–3, 12^, yet without a guarantee of success. This is of no surprise since finding the global minimum on a given potential-energy-surface, a key criterion for predicting ground state structure of a cluster, is essentially a formidable task. The number of local minima increases exponentially with the increasing cluster size^3, 6, 13^, as it is obviously an NP-hard problem that can be mapped to the traveling-salesman problem^13, 14^. In the past two decades, several global optimization methods for structure prediction have been devised^15^, including simulated annealing^16^, basin/minima hopping^17–19^, genetic algorithm^19–27^, particle swarm optimization algorithm^28–33^, etc. To investigate the structural evolution by global optimization, a large number of trial structures are to be generated at each generation. Consequently, it is highly desirable to develop some techniques to label the transient structures and to judge the similarity or congruency among the structures available at the intermediate stages, which serve to avoid futile, repetitious computation so as to effectively accelerate the search process^33^. A complete sampling of all the minima on a potential-energy-surface is simply impossible, but a high-throughput screening under restricted conditions can be still very helpful^6, 34^, for which the congruence check of clusters is a crucial prerequisite. In order to congruence check two distinct clusters, it needs to establish a one-to-one mapping, or correspondence, between their structural elements. The most straightforward way would be to compare the coordinates of the corresponding atoms in those two clusters^35^. This coordinate-based method is very complicated because both clusters are subjected to a translation operation so that their centroids are at the origin of the coordinate system, and one of them needs an additional rotation to minimize the deviation in the corresponding coordinates. For two 2D clusters each containing *n* atoms, the largest amount of comparing data this way is significantly greater than 2*n* pairs (for square lattice, without distinguishing the enantiomers, it is usually 16*n* pairs). An alternative strategy is to compare the corresponding interatomic distances in the two clusters, i.e., the distance-matrix method^30, 35^. However, as Lv *et al*.^33^ once pointed out, “the distance metric requires ordering of atoms in a structure, and thus is not able to unambiguously fingerprint structures”. For two clusters of size *n*, the largest amount of comparing data in this way is much more than *n*(*n* − 1)/2 pairs. Per-atom distance sets fingerprint and diffraction-like fingerprint recently proposed by Oganov and Valle^36, 37^, from which a reasonable measure of structure of similarity was derived, can be used to discriminate structures of a majority of clusters, but they cannot be used to label distinct structures since there are still some clusters cannot be distinguished through the two fingerprints, even combining both of them (an example is illustrated in the supplementary materials). Other methods such as those involving Bond-Characterization-Matrix^32, 33^, Zernike descriptors^38^, spherical harmonic descriptors^39^, can provide an approximate, quantitative measure for structural similarity between two clusters, but cannot label the distinct structures. The aforementioned methods are all suitable for both amorphous and crystalline clusters. For 2D and 3D high-symmetry crystal-fragment clusters, where the interatomic distances among the nearest neighboring atoms are fixed, there is also another recognition technique based on the relative orientations of the bonds, which employs codes obtained by using the Balaban and von Schleyer’s technique^40, 41^. In this approach the largest amount of comparing data is reduced to (*n* − 1) pairs, it is, however, not a fast structure retrieval method since finding the main and side chains of a given structure is very time-consuming.

In the current work we introduce a new indexing scheme, the minimum vertex-type sequence, to label and characterize the clusters on square lattice (below often simply referred to as cluster when no ambiguity may arise), which might meet some requirements for fast and effective decision making of artificial intelligence as in the game of Go^42^. This indexing scheme employs only the vertex type, or precisely the nearest-neighbor configuration, for each atom in a cluster. The atoms in a cluster are ordered and labeled following a special rule that the cluster can be indexed with a unique digit sequence, being independent from either the choice of the reference frame or the orientation of a cluster in given configuration. A one-to-one correspondence between the minimum vertex-type sequence and the cluster can be established. For clusters of size *n*, the largest amount of comparing data for congruency check is only *n* pairs.

## Results

Below we will demonstrate that for crystal-fragment clusters, the minimum vertex type sequence, which characterizes the nearest-neighbor configuration of each atom in a cluster, provides a practicable algorithm for fast structure comparison. The minimum vertex-type sequence method will be demonstrated in detail by treating clusters on square lattice.

**Figure 1:**
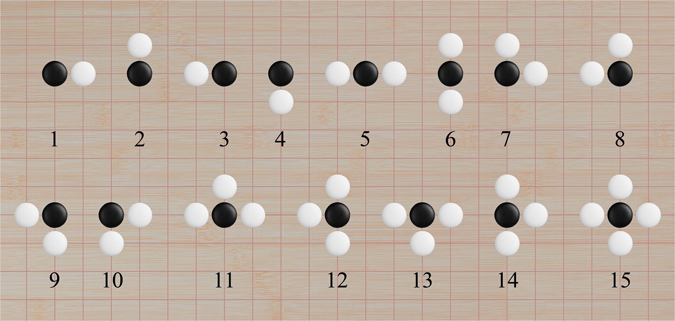
The fifteen possible vertex types appearing in a cluster on square lattice, as labeled by integers 1–15. The black stones denote the atoms of concern, while the white stones indicate the existence of possible nearest neighbors.

Firstly, we classify the vertexes for atoms in a cluster on square lattice based on their nearest-neighbor configurations (the orientations of the bonds with the nearest-neighbors are discriminated). There are only fifteen different vertex types for clusters on square lattice, which are labeled accordingly with the integers 1–15 in Fig. 1.

**Figure 2:**
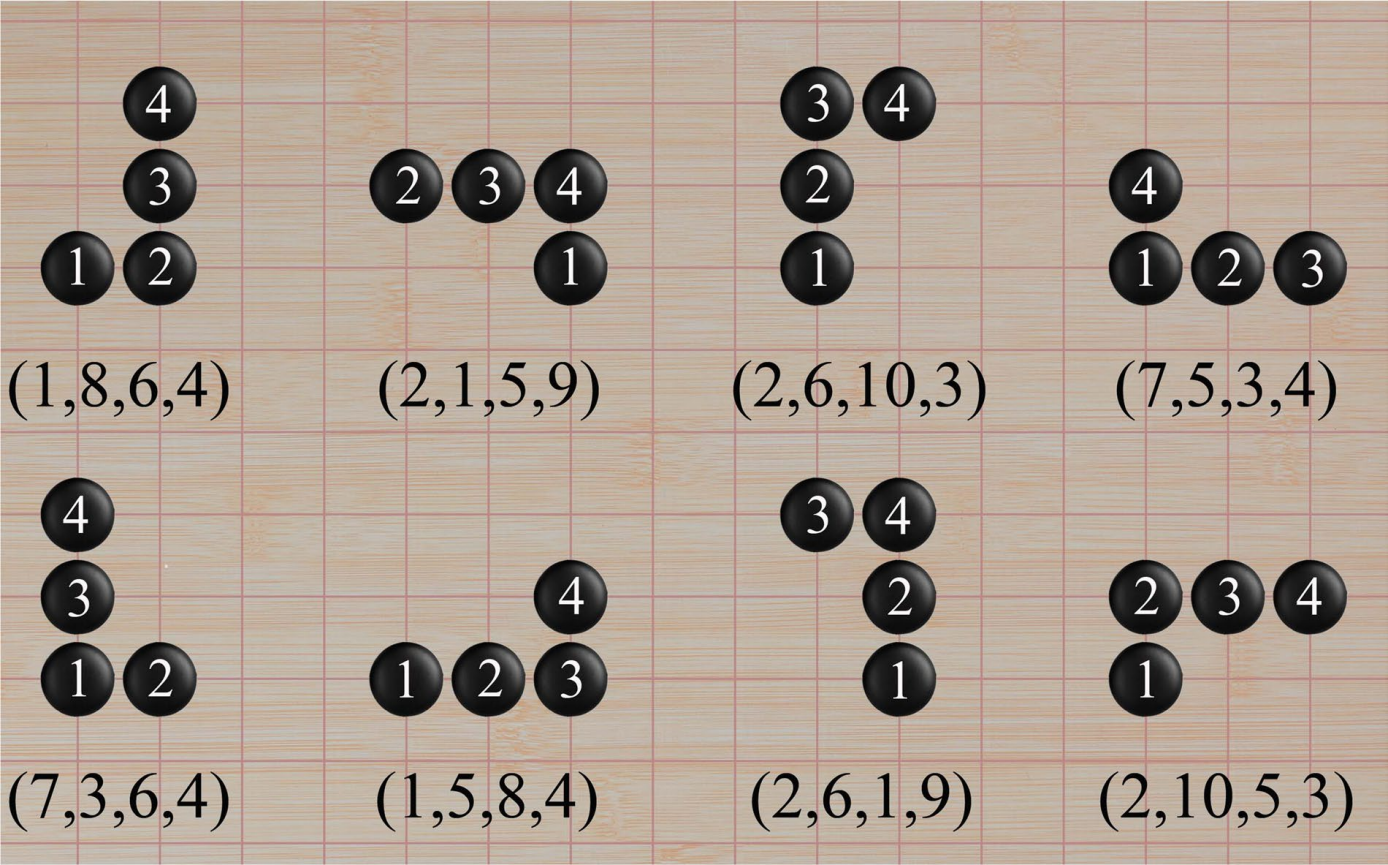
The different orientations of a given structure for 4-atom cluster under the action of group D_4_ for square lattice and the corresponding vertex-type sequences. The minimum vertex-type sequence is (1,5,8,4).

Secondly, atoms in the cluster are labeled with their vertex types in the order of “left-to-right-and-bottom-up”, i.e., the atom at the bottom left corner is set to be the first atom, it is then the turn of atoms in the same horizontal line from left to right till the end of that line, which is to be continued from the leftmost atom of the next upper line (if there is any). This process will proceed to the atom at the upper right corner of the cluster, as shown in Fig. 2.

By assigning to all the atoms a corresponding vertex type in the aforementioned order, we obtain an *n*-digit vertex-type sequence, where *n* is the size of the cluster. Since the vertex types are discriminated with regard to the orientations of bonds to the nearest-neighbors, the same cluster in different orientations may have distinct vertex-type sequences. As the space group for square lattice is group D_4_ with eight elements, a cluster here concerned may have at most eight different vertex-type sequences. In Fig. 2 displayed are the different configurations of a cluster of 4 atoms under the action of group D_4_, the eight vertex-type sequences are (1,8,6,4), (2,1,5,9), (2,6,10,3), (7,5,3,4), (7,3,6,4), (1,5,8,4), (2,6,1,9) and (2,10,5,3), respectively.

Among those eight (or less if the clusters possess rotation and/or reflection symmetries) vertex-type sequences, we adopt the one which has the minimum preceding numbers as the character of the cluster, and denote it as the minimum vertex-type sequence. For the cluster of 4 atoms shown in Fig. 2, the minimum number for the first digit is 1, and in the two sequences beginning with 1, i.e., (1,8,6,4) and (1,5,8,4), the minimum number for the second digit is 5, thus the minimum vertex-type sequence for this cluster is (1,5,8,4).

It can be strictly demonstrated that there is a one-to-one mapping between the minimum vertex-type sequence and the cluster (see the supplementary information for details). This is to say that the minimum vertex-type sequence can be used to characterize the individual clusters on square lattice, which can serve the identification and comparison of the clusters. With the minimum vertex-type sequence, the largest amount of data involved in the comparison of two clusters of *n* atoms can be reduced to only *n* pairs.

For the comparison of two clusters, this minimum vertex-type sequence scheme has two distinct advantages over the conventional coordinate-based methods. It uses only a few descriptive data and, more importantly, it is orientation independent. Unlike the distance-matrix method which suffers from ambiguous ordering of the atoms^33^, the labeling of atoms in the minimum vertex-type sequence is concise and determinate. In comparison with the Balaban and von Schleyer’s technique^40, 41^, our minimum vertex-type sequence scheme employs only one single type of data, and it avoids the difficult process of finding out the main chains of a cluster, which is usually a formidable task. Roughly speaking, our minimum vertex-type sequence scheme uses only a series of integers labeling the vertex type of atoms, whereas the Balaban and von Schleyer’s technique employs three types of data, i.e., digits denoting the orientation of the bonds, parentheses denoting the branches and commas denoting the different branches on the same level. An overview of the comparison of the minimum vertex-type sequence scheme to those conventional techniques is summarized in Table 1. Obviously, the minimum vertex-type sequence scheme can serve the fast congruence-check for clusters on high-symmetry lattices.

**Table 1:** Comparison of the four structural description schemes for clusters on high-symmetry lattice.

Method	Information	Orientation independence	Labeling of atoms	Number of data types	Complexity of data sampling	Data size
CB	coordinates	No	N/A	1	easy	*mn* ^a^
DM	distances	Yes	Ambiguous	1	easy	(*n* ^2^ − *n*)/2
BS	Bond orientations	Yes	definite	3	hard	*n* − 1^b^
MVTS	Vertex types	Yes	definite	1	easy	*n*

CB: coordinate-based method; DM: distance-matrix method; BS: Balaban & von Sheleyer’s technique; MVTS: minimum vertex-type sequence.

^a^ *m* denotes the dimension of the system concerned.

^b^If a structure is formed only from the main chain, the size of the descriptive data is (*n* − 1); if a structure is branched, the data size is then greater than (n − 1).

## Discussion

**Figure 3:**
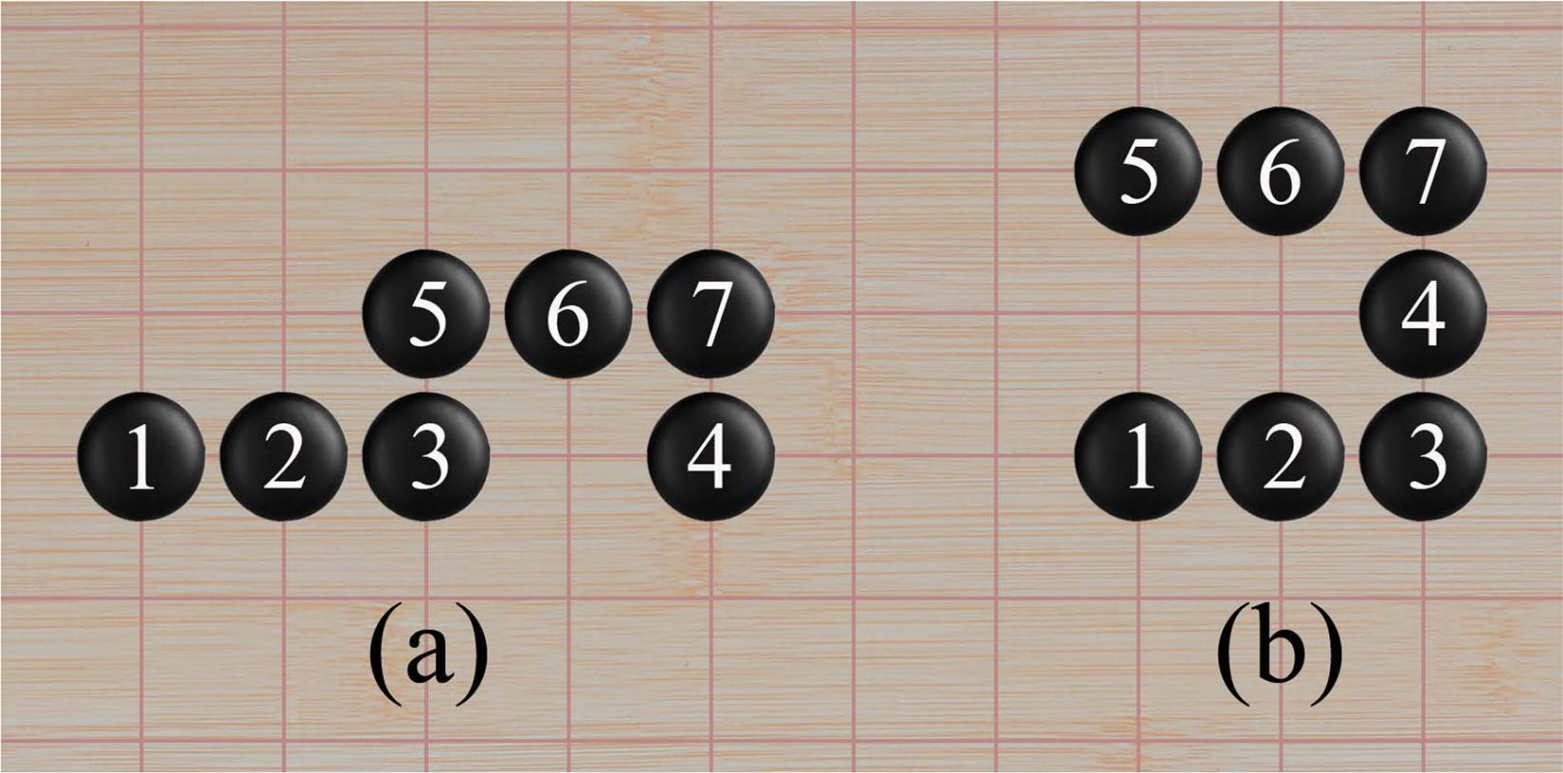
The two configurations for cluster of 7 atoms corresponding to the minimum vertex-type sequence (1,5,8,2,10,5,9) (**a**), and (1,5,8,6,1,5,9) (**b**), respectively.

The minimum vertex-type sequences build a one-to-one correspondence with clusters on square lattice, thus a minimum vertex-type sequence can be regarded as the index tag of the corresponding cluster. That is to say that each cluster can be characterized by a unique minimum vertex-type sequence. For example, the clusters of size 2 can be indexed as (1,3). The cluster of size 4 displayed in Fig. 2, which is one of the five possible configurations, can be indexed as (1,5,8,4). For clusters of size 7 in the two configurations in Fig. 3 (there are 108 different configurations in total), the minimum vertex-type sequences are (1,5,8,2,10,5,9) and (1,5,8,6,1,5,9), respectively. Comparing with the Balaban and von Schleyer’s indexing system^40, 41^, our minimum vertex-type sequence indexing system has at least two advantages. Firstly, the process of obtaining the minimum vertex-type sequence of a cluster is simpler than that to get the Balaban and von Schleyer’s codes which usually require the determination of the main and the side chain(s) in a branched cluster. Secondly, the minimum vertex-type sequence indexing system is more economical for computer calculation, since the minimum vertex-type sequences use only one type of data, whereas the Balaban and von Schleyer’s codes usually use three types of data.

In a practical implementation of congruence check for clusters of large sizes, approximate representation of single-number indexing can be used as a rapid primary filter, which is to be further confirmed by comparing the minimum vertex-type sequences. A good choice for the approximate representation of single-number indexing is to let $f(P)={\sum }_{i=1}^{n}{S}_{i}\cdot {i}^{6}$, where *S*_*i*_ is the *i*-th digit in the minimum vertex-type sequence of the cluster P. The final decision can be made by digit-by-digit comparing the corresponding minimum vertex-type sequences among the chosen candidates.

The minimum vertex-type sequence indexing scheme is orientation independent and employs only *n* integers to describe the configuration of a cluster on the square lattice. It is anticipated to provide a fast structure recognition method for large clusters. The excellent effectiveness of this indexing scheme can be verified in solving this typical problem: finding out all the isomers on square lattice for clusters as large as possible in a tolerable running time. This is a formidable task because the number of isomers increases exponentially with the cluster size^3, 6, 13^. When the cluster size reaches 13, as derived with the current method, there are 238591 types of isomers (with the enantiomers undistinguished). Each time when a new structure is generated, it needs to be compared with a great amount of existing isomers to judge whether a new type has occurred or not, which might immediately use up the computer resource. It is a difficulty for all structure recognition methods. And our minimum vertex-type sequence indexing scheme presented above provides a preferable fast structure retrieval method. Note that it is not time-consuming for a computer to label a cluster on square lattice using our minimum vertex-type sequence indexing scheme (say 1000 atoms, about 0.4 second on a Lenovo_C560, a general type of personal computer).

The minimum vertex-type sequence indexing also has many other merits. A minimum vertex-type sequence contains the information of vertex types of atoms, i.e., the nearest-neighbor configurations of atoms. It can be easily converted into the information of bonds. Therefore, the minimum vertex-type sequence indexing is preferable in the situation where the bond energy^4^ is of concern.

Furthermore, the concept of minimum vertex-type sequence can be generalized to other high-symmetry lattices, such as the regular triangular lattice and the honeycomb lattice, following three typical steps: 1) classify the possible vertexes in clusters on a given lattice based on the nearest-neighbor configuration of an atom; 2) label the atoms in the cluster with their vertex types in the order of “left-to-right-and-bottom-up”; 3) carry out all the possible symmetry operations of the given lattice on the cluster to obtain several vertex-type sequences among which we adopt the one with the minimum preceding numbers as the character of the cluster. Note that in the case of square lattice there are 15 possible vertex types to be handled, while there are 63 and 14 for the regular triangular lattice and the honeycomb lattice respectively (more details will be presented in a forthcoming publication). By combining the minimum vertex-type sequence method with high-throughput screening procedure under restricted conditions, effective strategies can be devised to search new atomic cluster structures on those complicated lattices.

We devised the minimum vertex-type sequence indexing scheme to characterize clusters on square lattice. The minimum vertex-type sequence of a cluster comprises only one integer for each atom to label its vertex type, and the sequence is orientation independent. It turns out to be the most preferable scheme for fast recognition and strict comparison of clusters, as it employs fewer and simpler descriptive data. In particular, for fast preliminary filtering, the minimum vertex-type sequences can be at first converted into a single integer, which can further accelerate the procedure of structure comparison dramatically. Our work is anticipated to initiate the search of other practical indexing schemes for handling clusters of large sizes, which are desperately desired in many fields. It is true that clusters and crystals usually contain bonds of different lengths, but they are beyond the scope of the manuscript. Our MVTS indexing scheme cannot deal with this case. Note that the minimum vertex-type sequence indexing does not provide a metric and is not capable of computing degrees of similarity, although it provides a high-efficient way for no-fail detection of equivalence of clusters comprised of crystal fragments. This is exactly complementary to the Oganov-Valle fingerprints, which offer a good pragmatic measure of structural similarity but may fail in some cases. Recently, another promising method is also provided by Oganov^43^ for improvement. The minimum vertex-type sequence indexing scheme would effectively speed up the ground state structure searching for the crystal-fragment clusters with large number of atoms.

## Electronic supplementary material

Supplementary Information
