# Supplementary material for: Minimum Vertex-type Sequence Indexing for Clusters on Square Lattice
Source: Sci Rep. 2017 Mar 24;7:392. doi: 10.1038/s41598-017-00398-z (PMC5428236; doi:10.1038/s41598-017-00398-z)
Supplement: Supplementary file 1 — Supplementary Information [file 41598_2017_398_MOESM1_ESM.pdf]

Supplementary Information

Minimum Vertex-type Sequence Indexing for Clusters on Square Lattice

Longguang Liao, Yu-Jun Zhao, Zexian Cao, and Xiaobao Yang

Contents

Ⅰ. Figures S1 and S2, and Table S1

Figure S1. Two distinct clusters, which cannot be discriminated from each other through Per-atom distance sets fingerprint and diffraction-like fingerprint.

Figure S2. The procedure of constructing a cluster according to the series of daughter sequences of a minimum vertex-type sequence.

Table S1. The corresponding data to the per-atom distance sets fingerprint of the two clusters illustrated in Supplementary Figure 1.

Ⅱ. An example demonstrating the fact that some clusters cannot be distinguished by Per-atom distance sets fingerprint and diffraction-like fingerprint

Ⅲ. Demonstration of the one-to-one correspondence between a cluster on square lattice and its minimum vertex-type sequence

References

Ⅰ. Figures S1and S2, and Table S1

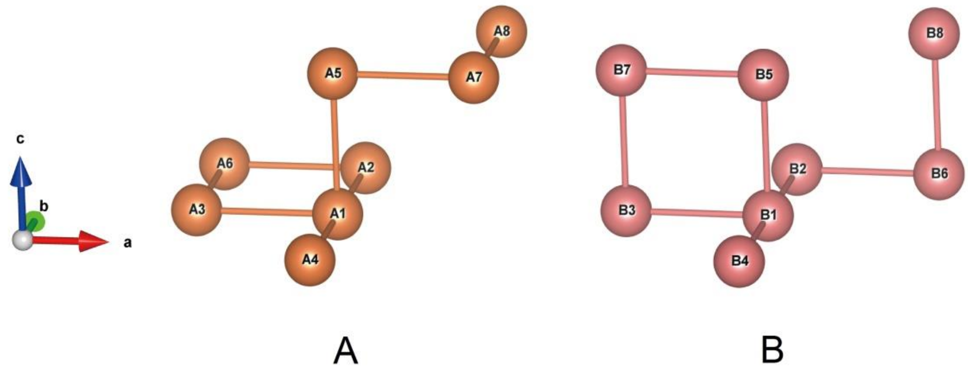

Figure S1. Two distinct clusters, which cannot be discriminated from each other through fingerprint methods of Oganov and Valle.

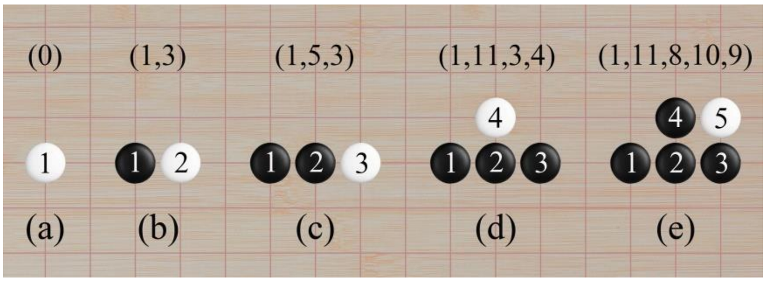

Figure S2. Constructing a cluster according to the series of daughter sequences of a minimum vertex-type sequence. The white stones indicate the newly added atoms at each step.

Table S1. The corresponding data to the per-atom distance sets fingerprint of the two clusters illustrated in Figure S1. [The per-atom distance sets fingerprint is composed by a section for each atom in the cluster (the unit cell in original text[1]). Each section is an ascending ordered set of distances from the corresponding atom to all the other atoms.]

A1(B1)	A2(B2)	A3(B3)	A4(B4)	A5(B5)	A6(B6)	A7(B7)	A8(B8)
1.0000	1.0000	1.0000	1.0000	1.0000	1.0000	1.0000	1.0000
1.0000	1.0000	1.0000	1.4142	1.0000	1.0000	1.0000	1.4142
1.0000	1.4142	1.4142	1.4142	1.4142	1.4142	1.4142	1.4142
1.0000	1.4142	1.4142	1.7321	1.4142	1.7321	1.7321	1.7321
1.4142	1.4142	1.4142	2.0000	1.4142	2.2361	1.7321	2.2361
1.4142	1.7321	2.2361	2.2361	1.4142	2.2361	2.2361	2.4495
1.7321	2.0000	2.4495	2.4495	1.7321	2.4495	2.4495	2.4495

Ⅱ. An example demonstrating the fact that some clusters cannot be distinguished by Per-atom distance sets fingerprint and diffraction-like fingerprint

The two fingerprints (per-atom distance sets fingerprint and diffraction-like fingerprint) proposed by Oganov and Valle[1, 2] can be used to discriminate configurations of a majority of clusters, but there are still some clusters cannot be distinguished from each other through the two fingerprints (even combining both of them). For example, the two clusters illustrated in Figure S1, whose coordinates are (0 0 0, 0 1 0, -1 0 0, 0 -1 0, 0 0 1, -1 1 0, 1 0 1, 1 1 1) and (0 0 0, 0 1 0, -1 0 0, 0 -1 0, 0 0 1, 1 1 0, -1 0 1, 1 1 1), respectively, cannot be distinguished from each other through the two fingerprints of Oganov and Valle (even combining both of them). These two clusters in Figure S1 may have the same per-atom distance sets fingerprint, whose data is shown in Table S1, and the same diffraction-like fingerprint. Therefore, fingerprint methods of Oganov and Valle (and the deduced distance measurement) cannot be used to label the distinct structures.

Ⅲ. Demonstration of the one-to-one correspondence between a cluster on square lattice and its minimum vertex-type sequence

In the main text it has been shown that a cluster can be converted into one and only one minimum vertex-type sequence. Here we need only to show that for a given minimum vertex-type sequence, one and only one cluster can be constructed thereupon.

The first item of business for us is to decompose a given minimum vertex-type sequence, i.e., to remove the digits one by one from the sequence till the initial digit of the original sequence. This procedure corresponds to the process of stripping off atoms from a cluster one by one till the original atom. Through this procedure, a series of daughter sequences with ever decreasing number of digits, and some of the remaining digits in the sequence have to be accordingly adjusted (see below), can be obtained. If it is always the last digit that is to be removed at each step, the thus derived series of daughter sequences is unique.

The second item of business is to construct the cluster by adding atoms one by one, based on the series of daughter sequences obtained in the foregoing decomposition process. It will turn out that the procedure of construction is also unique.

Decomposing a minimum vertex-type sequence

Before starting the decomposition procedure for a minimum vertex-type sequence, let’s first analyze the possible vertex types for the last atom of any clusters on square lattice.

It is easy to see that according to the "left-to-right-and-bottom-up" ordering rule, the possible vertex type for the last atom of a cluster on square lattice can be only one among the choices of 3, 4 and 9 (see Fig.1). This is to say that all the minimum vertex-type sequences must end with a digit of 3, 4 or 9 (Cf. Fig.2).

In the case (1) that the last atom P in the cluster is of vertex type 3, this atom P has only one nearest neighbor, the atom O, sitting directly left to atom P. Since there must be one atom sitting directly right to but without any atom sitting directly above it, the possible vertex types for the atom O can be only one among 1, 5, 10 and 13 (see Fig.1). Consequently, for a minimum vertex-type sequence ending with 3, the penultimate digit must be one among 1, 5, 10 and 13. After stripping off the last atom P of vertex type 3 from the cluster, the vertex type of the penultimate atom O will undergo the subsequent change: 5→3; 10→4; 13→9; and particularly 1→0. Here the auxiliary vertex type 0 refers to an isolated atom. An isolated atom, of vertex type 0, can appear in the residual fragments after stripping off atoms from a cluster of finite size.

In the case (2) that the last atom P is of vertex type 4, it has only one nearest neighbor atom, the atom Q, sitting directly below it. Having one atom directly above it, atom Q can assume one of the vertex types 2, 6, 7, 8, 11, 12, 14 or 15. It needs to point out here that among those atoms having a nearest neighbor directly above it, the atom Q is the last one in the cluster. As a result, in a minimum vertex-type sequence with the last digit being 4, there must be at least one digit being among the eight digits

2, 6, 7, 8, 11, 12, 14, and 15. Thus the last digit in the sequence being one among 2, 6, 7, 8, 11, 12, 14 and 15 refers to the atom Q in the cluster. After stripping off atom P of vertex type 4 from a cluster, the vertex type for the penultimate atom Q will consequently change this way: 6→4; 7→1; 8→3; 11→5; 12→9; 14→10; 15→13; and in particular 2→0.

In the case (3) that the last atom P is of vertex type 9, atom P has two nearest neighbors: One is the penultimate atom O sitting directly left to atom P, the other is atom Q sitting directly below atom P. The possible vertex types for atom O, as well as their transmutation after the stripping off of atom P, are just the same as in case (1) where the last atom P is of vertex type 3. The possible vertex types for atom Q, as well as their transmutation after the stripping off atom P, are just the same as in case (2) where the last atom P is of type 4. Note that the atom Q is the very last one among those atoms that may have a nearest neighbor sitting directly above it. Thus, for a minimum vertex-type sequence with the last digit being 9, the penultimate digit must be one among 1, 5, 10 and 13, and it must also contain at least one digit among the eight possibilities of 2, 6, 7, 8, 11, 12, 14 and 15. Any one of these eight possibilities that appears the latest in a minimum vertex-type sequence corresponds to atom Q.

In addition to the above three cases referring to vertex types 3, 4 and 9, there is another possibility for the last digit which is the ancillary vertex type 0 denoting an isolated atom. In this case, stripping off the atom referred to vertex type 0 does not affect the vertex type of any other atoms. Or we can say that by removing the last digit being 0 from a daughter sequence none of the other digits will be altered. Note that when there is only one digit left in a daughter sequence, this digit can be only 0.

Following the aforementioned digit-stripping rules, we can now decompose a given minimum vertex-type sequence by removing the digits one by one. Writing down the derived daughter sequence in each step, which has been partially altered with regard to the corresponding piece in the original minimum vertex-type sequence, we thus obtain a series of daughter sequences each with one less digit. For example, the minimum vertex-type sequence derived from the cluster shown in Fig.3a can be decomposed this way: (1,5,8,2,10,5,9) → (1,5,8,0,10,3) → (1,5,8,0,4) → (1,5,3,0) →

(1,5,3) → (1,3) → (0).

From the above discussion, we see that the process of decomposing a minimum vertex-type sequence is unique, since the derived series of daughter sequences refers to the process of stripping off atoms one by one from the cluster, and the series of the residual cluster fragments is unique.

Constructing cluster from a minimum vertex-type sequence

A unique cluster can be constructed based on the series of daughter sequences of a minimum vertex-type sequence. The correspondence between the n-digit minimum vertex-type sequence and the atom configuration in a cluster of size n is guaranteed, but not readily to be recognized. Fortunately, the decomposition of the minimum vertex-type sequence into the series of daughter sequences provides a step-by-step guidance for the construction of the cluster from scratch.

It begins with an isolated atom, of vertex type 0. This atom 1 can be placed on any lattice point on the square lattice. Without loss of generality, suppose it is put on the origin of the coordinate system for the square lattice. Then more atoms will be added one by one to the acquired structure. Below we will show that the relative position of the atom to be added at each stage can be determined by two successive daughter sequences previously obtained by decomposing a given minimum vertex-type sequence.

Let's consider the addition of the k-th atom to an existing cluster fragment of $(k-1)$ atoms, where k>1 is a positive integer. Four different situations may be confronted by this atom k.

In the case that the last digit is 3 in the k-digit daughter sequence, the atom k should be placed on the right side of the $(k-1)$-th atom. In this case, the k-digit daughter sequence differs from the (k-1)-digit daughter sequence only in its (k-1)-th digit, of course it now also has the additional k-th digit, as illustrated by panels a-b and panels b-c of Figure S2, respectively.

In the case that the last digit of the k-digit daughter sequence is 4, the atom k

should be placed directly above some g-th atom ($g < k$). To determine the value of g, we notice that the k-digit daughter sequence differs from the preceding $(k-1)$-digit daughter sequence only at the g-th digit, and has the additional k-th digit. For example, given that the successive daughter sequences of 3- and 4 digits be (1,5,3) and (1,11,3,4), respectively, we know that atom 4 should be placed directly above atom 2, as illustrated in panels c-d of Figure S2.

In the case that the k-digit daughter sequence terminates with 9, the atom k should be placed on the right-hand-side of the $(k-1)$-th atom. At the same time, it also should be directly above some g-th atom ($g < k-1$). To determine the value of g, we notice that the k-digit daughter sequence differs from $(k-1)$-digit daughter sequence at the g-th and $(k-1)$-th digits, and has the additional k-th digit. For example, given that the two successive sequences of 4 and 5 digits be (1,11,3,4) and (1,11,8,10,9), then atom 5 should be placed on the right hand side of atom 4 and meanwhile directly above atom 3, as shown in panels d-e of Figure S2.

In the particular case that the last digit of the k -digit daughter sequence is 0, the relative position of atom k with respect to the atoms in the existing fragment cannot be immediately determined, yet the problem can be finally settled on the basis of the whole series of daughter sequences because of the integrity of a cluster. To determine the position of the atom k, information from a few lengthy daughter sequences is demanded. In practice, we can proceed along the following line. The first atom of vertex type 0, i.e. atom 1, is placed at the origin. Atoms having a definite position relative to atom 1, including this atom 1, are grouped into branch one. The second atom of vertex type 0, labeled as atom B for the moment, will be temporarily placed at a point that is sufficiently distant to avoid any overlap between branch one and the forthcoming branch to grow around atom B. The forthcoming atoms having a definite position with regard to atom B are grouped into branch two. Other isolated atoms of vertex type 0, if there are any, and the forthcoming atoms, are handled in the same

way. When a freshly added atom connects two existing distinct branches, the atoms in the younger branch should be taken to merge into the elder one. At the final stage of construction, the cluster as an integral unity should have just one branch.

For example, let's construct a cluster from a given series of daughter sequences of (0) → (1,3) → (1,5,3) → (1,5,8,4) → (1,5,8,4,0) → (1,5,8,4,1,3) → (1,5,8,6,1,5,9) (for the decomposition procedure the series is presented in the reversed order). It proceeds as follows: Atom 1 is placed on the origin; atom 2 is placed directly right to atom 1; atom 3 is placed directly right to atom 2; and atom 4 is placed directly above atom 3. These four atoms have definite relative positions with respect to atom 1, and they are grouped into branch one having a size of 4. The last digit of the 5-digit daughter sequence is 0. Thus, atom 5 is the second atom of vertex type 0, it is temporarily placed at a faraway point to guarantee non-overlapping with branch one. Atom 6 is placed directly right to atom 5. So far, atoms 5 and 6 are grouped into branch two. Next we see that the atom 7 should sit directly above atom 4, and meanwhile right to atom 6—therefore the atom 7 connects the two branches. The thus obtained cluster is shown in Fig.3b.

From the above analysis we see that the relative position of each atom with respect to atom 1 can be determined on the basis of the series of daughter sequences obtained through the decomposition of a given minimum vertex-type sequence. It is definite and unique.

A true minimum vertex-type sequence for clusters on square lattice needs meet two requirements: (1) It can be decomposed into a series of daughter sequences each with one less digit; (2) A one-piece cluster can be constructed following the rules of construction on the basis of the series of daughter sequences. The one-piece cluster can be encoded into a minimum vertex-type sequence which reproduces the original series of daughter sequences. For such a minimum vertex-type sequence, the procedure for both decomposition and construction is unique.

References

1. Oganov, A.R. & Valle, M. Crystal Structures Classifier for an Evolutionary Algorithm Structure Predictor. *IEEE Symposium on Visual Analytics Science and Technology*, 11-18 (2008).

2. Oganov, A.R. & Valle, M. How to quantify energy landscapes of solids. *J. Chem. Phys.* **130**, 104504 (2009).
